# Tube Length Adjustment and Tube Trimming Technique in Refractory Glaucoma

**DOI:** 10.1155/2020/8889448

**Published:** 2020-06-22

**Authors:** Iwan Soebijantoro, Nina Asrini Noor

**Affiliations:** JEC Eye Hospital Kedoya, Jakarta, Indonesia

## Abstract

Secondary glaucoma may develop after vitreoretinal surgery as it is a known risk factor for its development. When the risk factors are more than one, for instance along with neovascular glaucoma (NVG), the secondary glaucoma may become recalcitrant and very difficult to manage. Surgical intervention is often warranted to control intraocular pressure (IOP) and prevent progressive glaucomatous damage in patients with refractory glaucoma, and glaucoma drainage implant may be preferred as the primary choice. We describe a patient who develop secondary glaucoma after vitrectomy and silicone oil (SO) injection due to unresolved vitreous hemorrhage in proliferative diabetic retinopathy (PDR) and subsequent NVG. Baerveldt glaucoma implant (BGI) was carried out and placed in the superotemporal quadrant with longer anterior chamber tube placement to prevent escape of SO through the tube. Qualified success was achieved with additional one fixed-drug combination (FDC). However, 3 years later, the tube was blocked by the iris tissue at the inferior edge of the pupil. Tube trimming was performed efficiently using a simple technique. The distal end of the tube was pulled out of the anterior chamber through a paracentesis just next to the tube entrance and trimmed to the appropriate length. More than a year after the surgery, IOP was still well controlled with the same FDC. Unfortunately, the visual acuity could not be recovered due to advanced PDR.

## 1. Introduction

Refractory glaucoma after vitreoretinal surgery is not rare occurrence. It has been reported in up to 30% of patients 2 years after vitrectomy with silicone oil (SO) injection [1]. It develops due to surgery and tamponade agents, is usually transient, and is generally managed with antiglaucoma therapy [2]. However, when it arises in conjunction with other form of secondary glaucoma, such as neovascular glaucoma (NVG), it may become very difficult to treat. Several factors were thought to play a role in the development of intraocular pressure (IOP) increase after vitreoretinal surgery, although they are not fully understood. Oxidative damage to the trabecular meshwork, secondary scarring, and SO complications were some of the possible mechanisms [3–9].

Glaucoma implants have become very useful for refractory glaucoma in the eyes with previous vitreoretinal surgery and NVG. Both nonvalved and valved implants have been used for the management of refractory glaucoma [7, 8]. Complications in tube shunt surgery are not uncommon, and one of them is tube occlusion which may require tube revision or trimming [9, 10]. However, in certain cases, tube trimming may be considered as major intervention with risk of further complications.

Here, we report the case of a patient who developed secondary glaucoma after vitrectomy surgery and SO injection for unresolved vitreous hemorrhage in proliferative diabetic retinopathy (PDR). The patient also developed NVG subsequently. Baerveldt glaucoma implant (BGI) was performed, and the tube was left longer in the anterior chamber near the inferior pupillary margin to avoid SO passing through the tube as SO removal was deemed not yet possible at that time. Despite being successful in reducing IOP along with one additional fixed-drug combination (FDC), the tube became occluded by the iris tissue three years later. This case briefly explains how we performed tube trimming with an effective and efficient approach. More than a year after the tube trimming, the IOP is under control while maintaining the same FDC.

## 2. Case Presentation

A 61-year-old male was referred to the glaucoma service of JEC Eye Hospital in September 2014. In the last one year, he was taken care by the retina service for PDR and diabetic macular edema (DME) from uncontrolled type II diabetes which he already had for 15 years. The patient had undergone multiple panretinal photocoagulation (PRP), intravitreal ranibizumab injections, cataract surgery with IOL implantation of both eyes, and subsequently vitrectomy and SO in August 2014 due to unresolved vitreous hemorrhage of the left eye.

The patient was referred to glaucoma service one month after vitrectomy and SO surgery due to uncontrolled IOP of the left eye despite medication with brimonidine tartrate 0.2% ophthalmic solution (Alphagan®, Allergan Inc., United States) and dorzolamide hydrochloride 2% eye drops (Trusopt®, MSD, United Kingdom) combined with oral extended release acetazolamide 500 mg capsule (Diamox® Sequels, Wyeth Pharmaceuticals Inc., Canada).

He presented with visual acuity 1-meter finger counting and IOP 37 mmHg. Iris neovascularization was markedly observed, and he was diagnosed with NVG as a secondary mechanism contributing to the IOP increase besides postvitrectomy and the presence of SO. The patient was immediately planned to undergo glaucoma implant surgery which took place a couple of days after his initial visit to glaucoma service.

**Figure 1:**
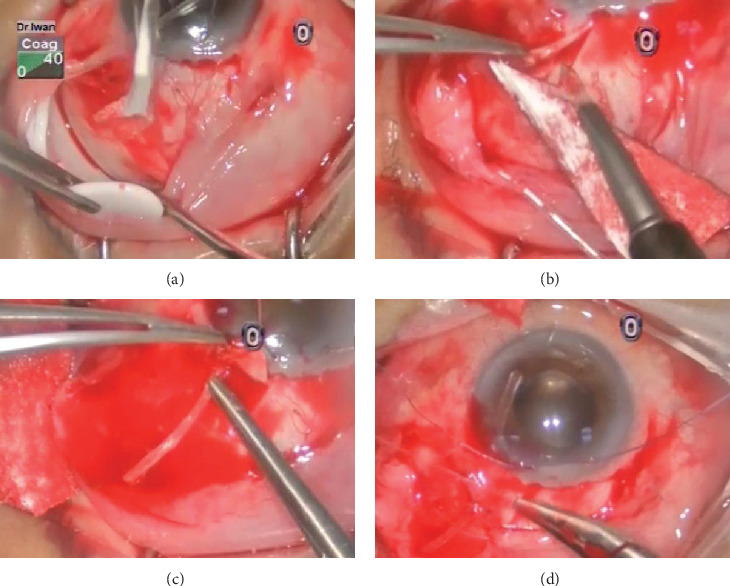
Summary of the Baerveldt glaucoma implant (BGI) surgery. (a) The implant was slipped between the sclera and Tenon's capsule 10 mm posterior to the limbus and then positioned and securely sutured at superotemporal quadrant. (b) A scleral flap was made for the insertion of the tube, and then, the tube was cut and beveled anteriorly. (c) An opening was made underneath the scleral flap, and the tube was inserted into the anterior chamber. (d) The final position of the tube in the anterior chamber was checked, and scleral flap was sutured, followed by closure of Tenon's capsule and conjunctiva.

**Figure 2:**
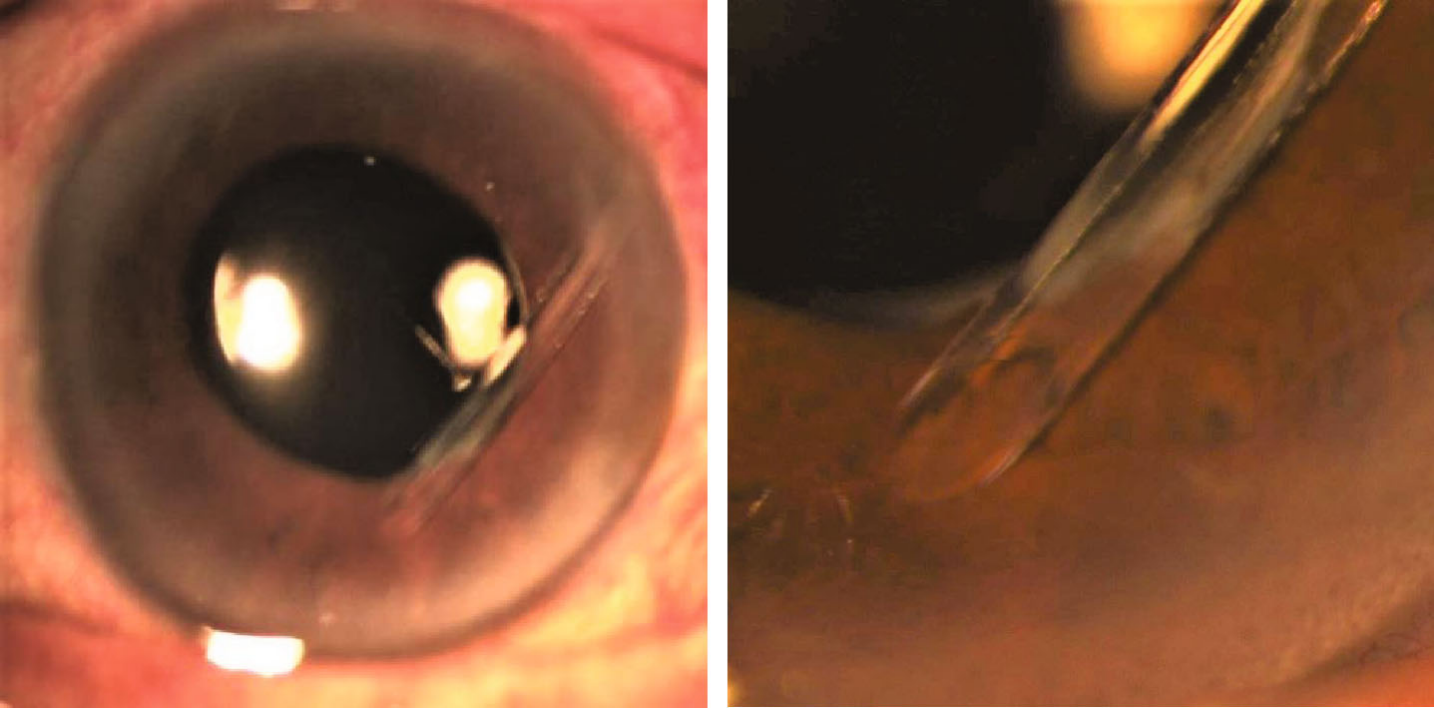
The final tube position after the initial BGI surgery. The tube was left longer in the anterior chamber just below the inferior pupillary margin.

The patient underwent an uncomplicated Baerveldt implant (model BG-101-350, Abbott Medical Optics, Santa Ana, California, United States) surgery (Figure 1). A 350 mm^2^ Baerveldt implant was placed in the superotemporal quadrant. A limbus-based conjunctival flap was dissected, and the implant was sutured securely to the sclera. Due to the presence of SO, the tube was left longer and lower in the anterior chamber below the inferior edge of the pupil (Figure 2). This approach was taken to avoid the escape of SO through the tube if it was left shorter.

One week after the surgery, IOP was markedly decreased to 6 mmHg. Since then, he was routinely followed up in glaucoma service every month together under the care of retina service. The patient underwent SO removal of the left eye in January 2015 and several more intravitreal ranibizumab injections of both eyes. During three years of follow-up, the IOP was maintained in the low teens for three years with additional topical FDC of brinzolamide 10 mg/ml and timolol 5 mg/ml ophthalmic suspension (Azarga®, Alcon Laboratories Inc., United States).

**Figure 3:**
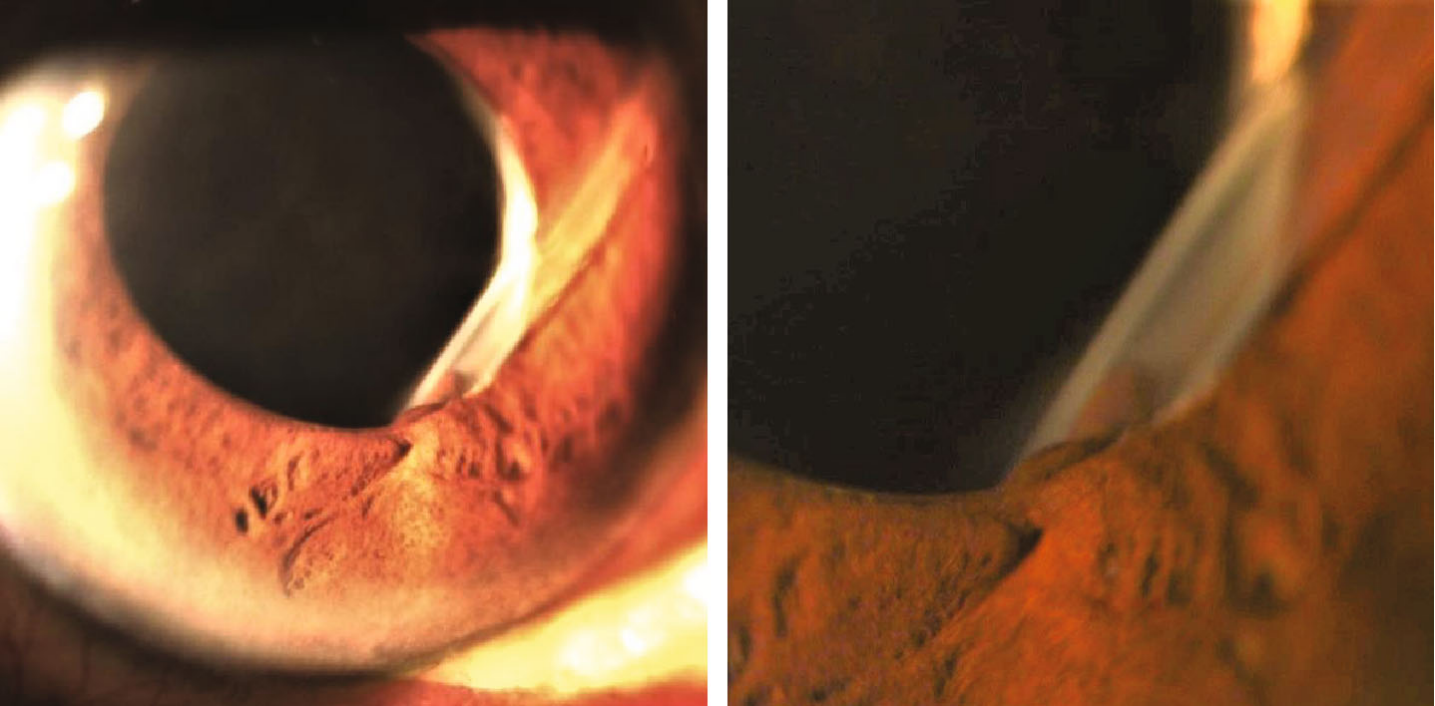
The tube was blocked by the iris tissue at the pupillary margin.

**Figure 4:**
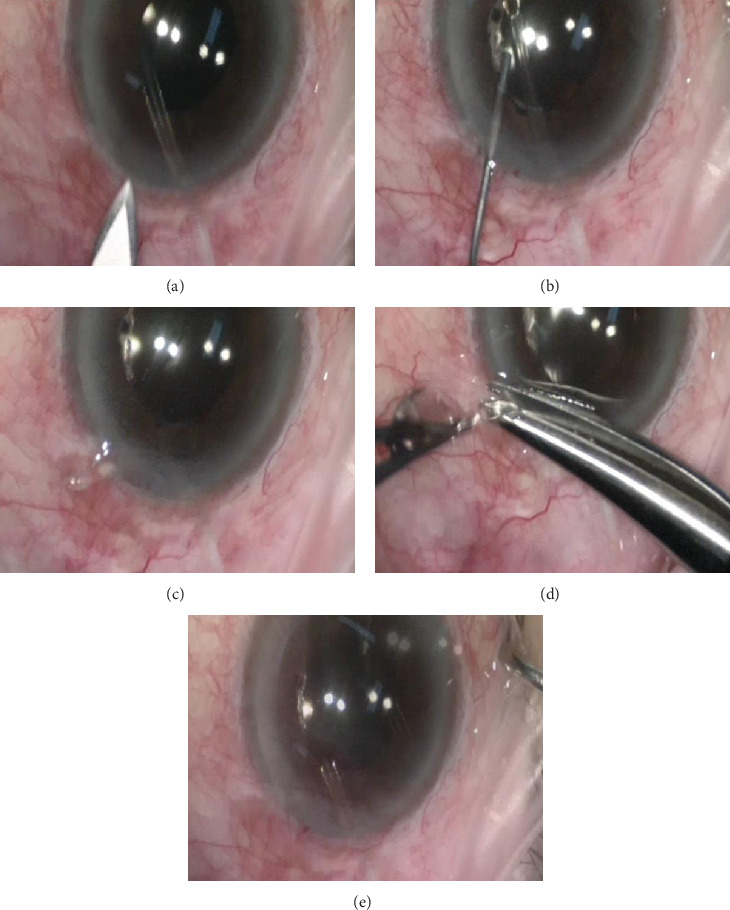
The tube trimming technique. (a) A single paracentesis was made approximately 1 clock hour temporally from the tube insertion. (b) Viscoelastic was injected to release the iris tissue blockage from the tip. (c) Through the paracentesis, the tube was pulled out from the anterior chamber using forceps. (d) The tube tip was held steady with forceps and transected with scissors. (e) Then, the tube was inserted back into the anterior chamber.

In February 2018, the IOP was suddenly elevated to 46 mmHg. From slit lamp examination, the cornea remained clear and there was no tube-corneal touch, but unfortunately, the tube was blocked by the iris tissue (Figure 3). Tube trimming was immediately performed efficiently with a relatively easy technique (Figure 4). A single paracentesis was made next to the tube insertion, followed by injection of viscoelastic to release the iris tissue which was blocking the tube tip. The distal end of the tube was then pulled out from the anterior chamber through the paracentesis using forceps. Next, the tube tip was transected with scissors for the desired length, beveled anteriorly, and subsequently inserted back into the anterior chamber.

One day after the surgery, IOP decreased to 11 mmHg and the tube was well-positioned near the superior pupillary edge. Thereafter, the patient was followed up every 2 months and maintained his previous FDC. Until his last visit in December 2019, the IOP was well-controlled in the low teens. However, the visual acuity was very poor and only hand movement due to the course of advanced PDR.

## 3. Discussion

Factors that play a role in the development of an IOP elevation after vitreoretinal surgery are not well understood. An increase in oxygen tension in the vitreous cavity after a vitrectomy, which is hypothesized to yield oxidative damage to the trabecular meshwork, is considered to play a role in the pathogenesis [3–6]. SO complications may also be responsible for IOP elevation after vitrectomy [11, 12]. Several mechanisms associated with SO-induced secondary glaucoma include pupillary block, inflammation, iris neovascularization, migration of emulsified and nonemulsified SO into the anterior chamber, and idiopathic open-angle glaucoma [1, 7].

It has been reported that 7.1–10% of patients who underwent vitrectomy and SO injection developed uncontrolled glaucoma despite medical therapy and removal of SO [13]. Eyes with an early postvitrectomy IOP elevation were more likely to have a persistent IOP increase for at least 6 weeks [12]. In this case, we observed an increase of IOP in the first week after vitrectomy surgery; thus, it was very likely that IOP would remain uncontrolled for longer period as also observed in the previous study.

NVG also played a significant role in this case. Severe PDR is a significant predictor of NVG [14]. In vitrectomized eyes, it is assumed that NVG can be more significant because vasoformative factors in the vitreous cavity could easily diffuse into the anterior chamber [15], although the presence of silicone oil may have a role in inhibiting progressive neovascularization in the anterior segment by preventing the diffusion of angiogenic substances [16]. In the end, the concurrence of NVG along with secondary glaucoma postvitrectomy and silicone oil in this patient contributed to an uncontrolled IOP despite maximum medications.

Refractory glaucoma in this case indicated the need for surgical treatment. Glaucoma implants have become very useful and may be preferred as the primary choice for refractory glaucoma in the eyes with previous vitreoretinal surgery and NVG with either valved or nonvalved implants [7, 8]. In Indonesia, the most commonly used implants are the Ahmed-184 and Baerveldt-350 implant with satisfying results, correlating with the results of studies in other Asian and non-Asian groups [9, 17, 18]. Baerveldt implant was used in a greater number of poor prognosis glaucoma eyes with higher IOP and in the more severe glaucoma cases, including NVG and postvitrectomy surgery [9]. For this rationale, Baerveldt implant was chosen and an anterior chamber tube was placed as pars plana tube placement was not an option due to the presence of silicone oil in the vitreous cavity. Longer tube was placed to anticipate the risk of migration of SO particles through the tube which may cause tube blockage or conjunctival fibrosis.

Complications in tube shunt surgery are not uncommon and may include tube exposure, tube occlusion, endophthalmitis, dysesthesia, uncontrolled IOP, low IOP, and diplopia [9, 10]. In this case, patient had tube blockage by the iris tissue approximately 3 years after the initial BGI surgery which certainly required trimming of the tube. Tube trimming is considered as major surgery and typically involves dissection through a scarred conjunctiva, dissection underneath the scleral patch graft, removal of the tube from the anterior chamber, trimming of the tube, and subsequent reinsertion and suturing. This process can be lengthy and may be fraught with complications.

Asrani et al. [19] demonstrated a case with newer technique for glaucoma tube trimming. In their paper, they explained a technique using two paracentesis, a 30-gauge needle to hold still the distal end of the tube through one paracentesis, a manual membrane peeling and cutting scissors to transect the tube through the second paracentesis, and finally a forceps to grasp and remove the transected part of the tube. Despite being a great technique, it requires multiple paracentesis and instruments. We performed a different approach which we felt easier, more efficient, and effective to perform by pulling the distal end of the tube out of the anterior chamber through a paracentesis, transecting the tube, and inserting it back into the anterior chamber. The tube was trimmed so that it was beveled anteriorly to prevent recurrent blockage by the iris tissue, as also suggested by Wang and Barton [20].

In a series by van Aken et al. [7], the majority of eyes with refractory glaucoma after vitrectomy still required glaucoma medication following BGI, and only 23% did not. Thus, the need of additional medication to control the IOP after surgery was anticipated in this patient.

BGI in combination with FDC glaucoma medication was successful in controlling the IOP in the low teens in this patient with refractory glaucoma. The length and position of the tube in the initial surgery should be tailored case-by-case. If tube trimming is necessary, a simple yet efficient technique described previously may be considered.

## Data Availability

The data used to support the findings of this study are available from the corresponding author upon request.
